# Phosphorylated recombinant HSP27 protects the brain and attenuates blood-brain barrier disruption following stroke in mice receiving intravenous tissue-plasminogen activator

**DOI:** 10.1371/journal.pone.0198039

**Published:** 2018-05-24

**Authors:** Yoshiaki Shimada, Hideki Shimura, Ryota Tanaka, Kazuo Yamashiro, Masato Koike, Yasuo Uchiyama, Takao Urabe, Nobutaka Hattori

**Affiliations:** 1 Department of Neurology, Juntendo University Urayasu Hospital, Chiba, Japan; 2 Institute for Environment and Gender Specific Medicine, Juntendo University School of Medicine, Chiba, Japan; 3 Department of Neurology, Juntendo University School of Medicine, Tokyo, Japan; 4 Department of Cell Biology and Neuroscience, Juntendo University School of Medicine, Tokyo, Japan; 5 Department of Cellular and Molecular Neuropathology, Juntendo University Graduate School of Medicine, Tokyo, Japan; Massachusetts General Hospital/Harvard Medical School, UNITED STATES

## Abstract

Loss of integrity of the blood-brain barrier (BBB) in ischemic stroke victims initiates a devastating cascade of events causing brain damage. Maintaining the BBB is important to preserve brain function in ischemic stroke. Unfortunately, recombinant tissue plasminogen activator (tPA), the only effective fibrinolytic treatment at the acute stage of ischemic stroke, also injures the BBB and increases the risk of brain edema and secondary hemorrhagic transformation. Thus, it is important to identify compounds that maintain BBB integrity in the face of ischemic injury in patients with stroke. We previously demonstrated that intravenously injected phosphorylated recombinant heat shock protein 27 (prHSP27) protects the brains of mice with transient middle cerebral artery occlusion (tMCAO), an animal stroke-model. Here, we determined whether prHSP27, in addition to attenuating brain injury, also decreases BBB damage in hyperglycemic tMCAO mice that had received tPA. After induction of hyperglycemia and tMCAO, we examined 4 treatment groups: 1) bovine serum albumin (BSA), 2) prHSP27, 3) tPA, 4) tPA plus prHSP27. We examined the effects of prHSP27 by comparing the BSA and prHSP27 groups and the tPA and tPA plus prHSP27 groups. Twenty-four hours after injection, prHSP27 reduced infarct volume, brain swelling, neurological deficits, the loss of microvessel proteins and endothelial cell walls, and mortality. It also reduced the rates of hemorrhagic transformation, extravasation of endogenous IgG, and MMP-9 activity, signs of BBB damage. Therefore, prHSP27 injection attenuated brain damage and preserved the BBB in tPA-injected, hyperglycemic tMCAO experimental stroke-model mice, in which the BBB is even more severely damaged than in simple tMCAO mice. The attenuation of brain damage and BBB disruption in the presence of tPA suggests the effectiveness of prHSP27 and tPA as a combination therapy. prHSP27 may be a novel therapeutic agent for ischemic stroke patients whose BBBs are injured following tPA injections.

## Introduction

Heat shock protein 27 (HSP27) is a small, highly conserved protein with various functions, including as a molecular chaperone, free radical scavenger, and anti-apoptotic factor, that interacts with cytochrome c and interferes with caspase activation complexes [1]. It is also known to act as a stabilizer of actin and microtubules of the cytoskeleton [2]. There is accumulating evidence that HSP27 protects various organs against stress [3]. HSP27-transgenic models exhibit numerous cytoprotective effects in *in vivo* models of various diseases, including cardiac ischemia [4,5], kainate-induced hippocampal cell death [6], nerve injury [7], the tau model of Alzheimer’s disease [8], [9], and the SOD1G93A model of amyotrophic lateral sclerosis [10]. HSP27 has also cytoprotective effect in ischemic stroke. HSP27-transgenic mice exhibit reduced infarcts after transient cerebral ischemia [11]. Badien et al showed that viral delivery of HSP27 and intraperitoneal injection of PEP1-HSP27 into ischemic animal models are also protective [12,13]. HSP27 also has cytoprotective effect in ischemic stroke. HSP27-transgenic mice exhibit reduced infarcts after transient cerebral ischemia [14]. Badien et al showed that viral delivery of HSP27 and intraperitoneal injection of PEP1-HSP27 into ischemic animal models are also protective [15,16]. Oligomerization and phosphorylation of HSP27 are both required for neuroprotection against ischemic neuronal injury in HSP27 transgenic mouse models [17]. We previously demonstrated that intravenously injected phosphorylated HSP27 protected the brains of mice with transient middle cerebral artery occlusion (tMCAO) [18,19], whereby it suppressed apoptotic cell death, oxidative DNA damage, and inflammatory responses, and also preserved the BBB.

The BBB maintains the movement of molecules, ions, and cells between neural cells and the blood, and regulates ionic homeostasis, hormonal and transmitter levels, and the transport of nutrients in the brain [20]. Its destruction, therefore, is one of the most disabling consequences of stroke [21]. Increased BBB permeability, matrix metalloproteinases, and aquaporins [22] lead to severe brain edema [22,23], a predictor of poor neurological outcome. Protection against these damaging sequelae of stroke may save human lives. Thus, maintenance of BBB integrity following stroke is a critical therapeutic necessity and deserves detailed investigation.

Recombinant tissue plasminogen activator (tPA) is the only effective fibrinolytic treatment at the acute stage of ischemic stroke; however, it is associated with an increased risk of brain edema and secondary hemorrhagic transformation. Thus, both cerebral ischemia and tPA treatment injure the BBB, and ischemic stroke patients treated with tPA require drugs to protect the BBB. Currently, only edaravone, a potent free-radical scavenger, is available for BBB protection [24–26]; however, it has limited use due to side effects, including liver and renal dysfunction [27].

Hence, a novel drug to protect the BBB is required for patients with ischemic stroke to prevent the expansion of ischemic damage, tPA-induced BBB injury, and hemorrhagic transformation. A drug is also required that can attenuate ischemic brain damage even when the BBB is injured. In this study, we tested the hypothesis that prHSP27 can attenuate ischemic brain damage and BBB disruption in BBB-injured tMCAO mice. We used hyperglycemia [28,29], tPA, and ischemic insults to injure the BBB. Pre-ischemic, hyperglycemic mice exhibit larger ischemic infarct volumes and more severe hemorrhage than normoglycemic mice [30], and this model has been widely used as a BBB injury model in various studies [28,29]. We examined whether intravenously injected prHSP27 attenuated brain damage and BBB disruption in tPA-injected, hyperglycemic tMCAO mice, whose BBB was more severely disrupted than that of tMCAO mice.

## Materials and methods

### Phosphorylated recombinant HSP27 preparation

rHSP27 (Acris Antibodies GmbH, Herford, Germany) was phosphorylated *in vitro* to form prHSP27 using recombinant human active MAPKAP kinase 2 (R&D Systems, Inc., Minneapolis, MN, USA) as described previously [18].

### Animals

All mouse procedures were approved by the Animal Care Committee of the Juntendo University. A total of 104 adult, 10-week-old, male C57BL/6 mice weighing 20–25 g were used in this study. All mice were housed under controlled lighting and provided with food and water *ad libitum*. The mice were anesthetized with 4.0% isoflurane (Abbott Japan Co., Ltd., Tokyo, Japan) and maintained on 1.0–1.5% isoflurane in 70% N_2_O and 30% O_2_ using a small-animal anesthesia system. The mice were subjected to 1-h tMCAO followed by reperfusion as described previously [31]. To monitor cortical perfusion, laser Doppler flowmetry was performed with the Periflux System (PERIMED, Inc., Stockholm, Sweden) before, during, and after occlusion by placing a probe on the skull at the midpoint between the right eye and right ear. Body temperature was maintained at 37 ± 0.5°C with a warming blanket during surgery. Mice showing occlusion (<20% of baseline) during ischemia and reperfusion (>80% of baseline) after the suture was withdrawn were included in the study (n = 104). 17% of the mice (n = 21) were excluded from the experiments. These mice were anesthetized with an intraperitoneal injection of 50 mg/kg pentobarbital and decapitated.

### Experimental protocol

To increase the BBB disruption and the occurrence of hemorrhagic transformation, D-glucose (6 mL/kg at 50% wt/vol) was injected intraperitoneally 15 min before tMCAO [32]. The mice were subjected to 1-h MCAO and then reperfusion. After 2 h of reperfusion, tPA (tPA; 10 mg/kg dissolved in PBS) (Activacin; Kyowa Hakko Kirin Corporation, Tokyo, Japan.), tPA (10 mg/kg) plus prHSP27 (50 μg/mouse dissolved in PBS), prHSP27 (50 μg/mouse), or bovine serum albumin (BSA; 50 μg/mouse) was injected via the tail-vein [18,19]. BSA had no influence in our study, and thus the BSA group served as a control.

During this procedure, body temperature was maintained at 37.0 ± 0.5°C with a heating pad. Twenty-four hours after reperfusion, the mice were anesthetized with an intraperitoneal injection of 50 mg/kg pentobarbital and decapitated. All subsequent assays were performed blinded to the mouse treatment group.

### Assessment of hemorrhagic transformation

To analyze and macroscopically classify the hemorrhagic changes, we stained coronal brain sections with 2,3,4-triphenyl tetrazolium chloride (TTC). Twenty-four hours after reperfusion, the mice were anesthetized with pentobarbital (50 mg/kg, i.p.) and transcardially perfused with 0.1 M phosphate buffer pH 7.4. Brains were removed, cut into 1-mm-thick coronal slices, and stained with 1% TTC in 0.1 M phosphate buffer. Using digital images, hemorrhages were classified into 5 groups based on type and extension as described previously: Grade 0: no hemorrhage; Grade 1: hemorrhagic infarction defined as small *petechiae*, generally along the boundary of the infarct; Grade 2: hemorrhagic infarction with more confluent *petechiae* within the damaged area; Grade 3: parenchymal hemorrhage characterized by blood clots in less than 30% of the injured parenchyma; and Grade 4: parenchymal hemorrhage with clots in more than 30% of the infarct [33].

### IgG staining

Twenty-four hours after reperfusion, mice were anesthetized with pentobarbital (50 mg/kg, i.p.) and decapitated. The brains were carefully removed and fixed in 4% paraformaldehyde for at least 2 days at 4°C and then overnight in 30% sucrose. To evaluate the permeability of the BBB to endogenous IgG proteins, we assessed coronal cryostat brain slices (20 μm) that were stained with IgG antibodies (donkey anti-mouse IgG, 1:300; Vector Laboratories). Immunoreactivity was visualized using the avidin–biotin complex method (Vectastain ABC kit, dilution 1:400; Vector Laboratories) [34], scanned with AxioVision software (Carl Zeiss MicroImaging GmbH, Göttingen, Germany), and measured using the ImageJ program (NIH, http://rsb.info.nih.gov/nih-image/). We measured the total luminances of the ipsilateral and contralateral hemispheres from each slice, and then computed an average luminance for each mouse. The number of mice in each treatment group are indicated for each result. IgG staining was then calculated as follows: (average of total ipsilateral hemispheric luminance − average of blank luminance) / (average of total contralateral hemispheric luminance − average of blank luminance).

### Measurement of serum glucose levels

D-glucose (6 mL/kg at 50% wt/vol) was injected intraperitoneally. Serum glucose was measured from the ophthalmic venous plexus before intraperitoneal injections (pre) and 15 min, 1 h, 2 h, 3 h, and 4 h after intraperitoneal injections.

### Measurement of infarct area and volume and brain swelling

To evaluate infarct area and volume, nine consecutive coronal cryostat brain slices (20-μm sections at 40-μm intervals) from the frontal cortex to the posterior striatum (forebrains) were stained with cresyl violet, scanned with AxioVision software (Carl Zeiss MicroImaging GmbH, Göttingen, Germany), and measured using the ImageJ program (NIH, http://rsb.info.nih.gov/nih-image/). We measured infarct volume for each brain was calculated by summation of the infarcted area of all slices. Brain swelling was calculated as follows: (total ipsilateral hemispheric volume × total contralateral hemispheric volume) / total contralateral hemispheric volume x 100). In addition, infarct volume corrected for edema was calculated as follows: (1 / 1 + brain swelling) × infarct volume.

### Neurological severity score

Neurological functions were evaluated a few minutes post MCAO and again 24 hours following reperfusion. Neurologic severity scores were based on motor, sensory, and reflex tests. These tests are similar to contralateral neglect testing described in humans [35]. The severity score of injury was graded on a scale of 0 to 14 (normal score 0, maximal deficit score 14). One point was awarded either for the inability to perform, or for abnormal task performance, or for the lack of a tested reflex. Each mouse was subjected to three rounds of each test. Observers of the behavioral test were blinded to the treatment group.

### SDS-PAGE gelatin zymography

Gelatin zymography was used to measure the levels of matrix metallopeptidase 9 (MMP-9) as described previously [36]. Twenty-four hours after reperfusion, the mice were anesthetized with pentobarbital (50 mg/kg, ip.) and transcardially perfused with 0.1 M phosphate buffer pH 7.4. The brains were quickly removed, divided into ipsilateral ischemic hemispheres and contralateral non-ischemic hemispheres, and the ischemic hemisphere of each mouse was homogenized in 10 volumes of lysis buffer (50 mmol/L Tris-HCl pH 7.4 containing 150 mmol/L NaCl, 1% IGEPAL CA-630, 0.1% SDS, and 0.1% deoxycholic acid) including protease inhibitors (2 μg/mL leupeptin, 2 μg/mL aprotinin, and 1 mmol/L phenylmethylsulfonyl fluoride) on ice with a Teflon glass homogenizer. After centrifugation at 10 000 × *g* for 7 min at 4°C, the supernatant was collected.

Protein concentrations were determined using a BCA protein assay kit (Thermo Scientific, Rockford, IL, USA). The samples (12 μg/lane) were prepared using a gelatin zymography kit (Cosmo Bio Co., Tokyo, Japan). Gels were scanned and analyzed with a standard computer-assisted imaging system. Due to the limit of electrophoresis gel size, we could not run all the samples on a single gel. Therefore, we used 3 gels after adjusting the protein concentration of the samples to load equal amounts of protein across the gels. We present the quantitative data on ratios of tPA and tPA+PrHSP27 to BSA after setting the area obtained from the BSA sample to 1.

### SDS-PAGE and immunoblotting

Proteins were separated by SDS-PAGE gels and subsequently transferred to a PVDF membrane. Block Ace (Daiichi Kogyo Seiyaku, Co., Ltd., Gifu, Japan) or phosphate-buffered saline (PBS) containing 0.05% Tween-20 (Sigma-Aldrich Co.) was used for blocking. PVDF membranes with transferred proteins were incubated overnight with antibodies against occludin (1:500; Invitrogen, Thermo Scientific^™^, Carlsbad, CA, USA). Immunoreactive bands were visualized with an enhanced chemiluminescence kit (GE Healthcare Life Sciences). Protein loading of lanes was assessed with α-tubulin immunoreactivity (53 kDa).

### Immunohistochemistry

Immunohistochemistry was performed on 20-μm, free-floating sections. Sections of forebrain and basal ganglia were stained overnight using rabbit anti-ionized calcium binding adapter molecule-1 (Iba-1; 1:500; Wako Pure Chemical Industries, Ltd., Osaka, Japan) and rabbit anti-collagen IV polyclonal (1:200; Abcam, Cambridge, UK) antibodies. The sections were then incubated with biotinylated secondary antibodies (1:300; Vector Laboratories, Inc.) and subsequently processed with avidin-biotinylated peroxidase complex (VECTASTAIN ABC Kit; 1:400; Vector Laboratories, Inc.).

### Lectin staining

To detect endothelial cells, 20-μm sections were incubated with 5 ug/mL FITC-labeled tomato lectin (*Lycopersicon esculentum*) (Vector Laboratories, Inc.), which recognizes endothelial cell walls, for 4 h at 4°C. Cellular nuclei were stained with DAPI. The fluorescence images were observed by a Keyence microscope. The volume of FITC labeling in the penumbra and ischemic core was computed using the image analysis software supplied with the microscope (Keyence, Osaka, Japan). Total FITC signal intensity was counted in 3 sections of penumbra area/mouse.

### Cell counts and statistical analysis

In the immunohistochemical analyses, positively stained cells in the ischemic boundary zone adjacent to the ischemic core (0.25 mm^2^), which is about 50 μm away from the infarct area [19], and basal ganglia in five sections from each mouse were counted using AxioVision. Blood vessels were identified by their morphology, and staining intensity (collagen IV) was measured using the ImageJ program (NIH, http://rsb.info.nih.gov/nih-image/). All values are expressed as mean ± standard error of the mean (SEM). One-way analysis of variance and post hoc Fisher’s protected least significant difference tests were used to determine the significance of differences between the groups. *P* values less than 0.05 indicated significant differences.

### Electron microscopy

Anesthetized rats were transcardially perfused with 2% paraformaldehyde/2% glutaraldehyde buffered with 0.1 mol/L phosphate buffer. Brain tissues were quickly excised and subsequently immersed in the same fixative overnight at 4°C. Using Rodent Brain Matrices (ASI Instruments, Warren, MI, USA), 1-mm-thick brain slices were made through the chiasm. Samples were harvested from the peri-infarct cortex area, post-fixed with 2% OsO_4_ in 0.1 mol/L phosphate buffer for 2 h at 4°C in the dark, block stained in 1% uranyl acetate for 1 h, dehydrated with a graded series of aqueous alcohol solutions, and embedded in Epon 812 (TAAB, Berkshire, UK). Silver sections were cut with an ultramicrotome (Leica UC6; Leica Microsystems, Vienna, Austria), stained with uranyl acetate and lead citrate, and observed with an electron microscope (HT7700; Hitachi, Tokyo, Japan).

## Results

### Effect of treatment with prHSP27 with or without tPA on infarct volume and neurological deficit in mice with tMCAO receiving tPA

**Fig 1:**
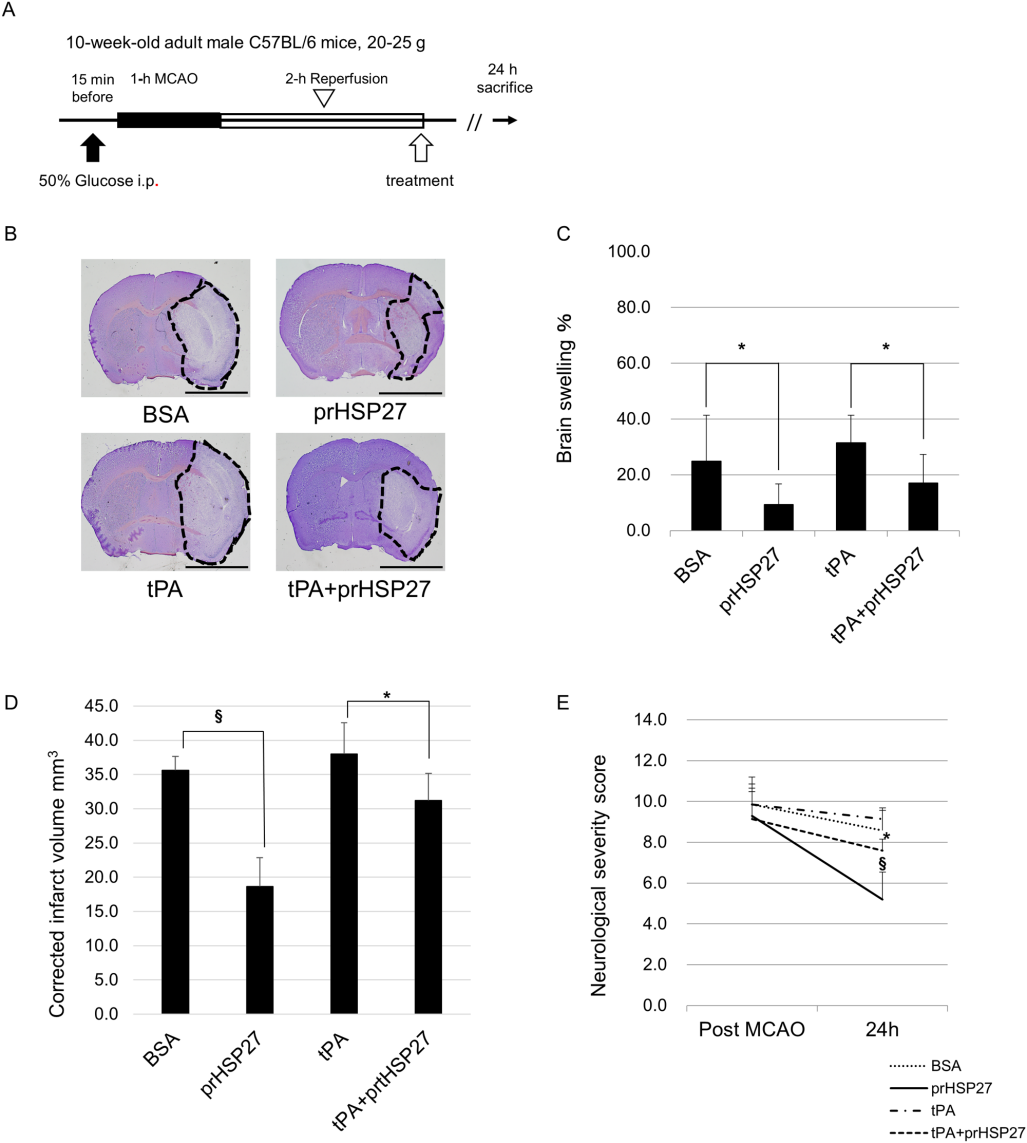
Experimental protocol. (A) D-glucose was injected intraperitoneally at 15 min before 1-h transient middle cerebral artery occlusion (MCAO). Following MCAO, the mice were re-perfused for 2 h and then injected with recombinant tissue plasminogen activator (tPA, *n* = 7), phosphorylated recombinant heat shock protein 27 (prHSP27, *n* = 6), tPA plus prHSP27 (tPA+prHSP27, *n* = 7), or bovine serum albumin (BSA, *n* = 9). The mice were sacrificed 24 h later. (B) Representative photomicrographs of infarct areas stained with cresyl violet at 24 h after reperfusion. Infarct areas are circumscribed with dotted lines. prHSP27 decreased brain infarct size. Scale bar = 2 mm. (C) Brain swelling. (D) Infarct volume corrected for edema. *p < 0.05, §*p* < 0.001. (E) prHSP27 improved neurological severity scores at 1 h following MCAO and at 24 h following reperfusion in mice treated with the above substances. Data are mean ± standard error of the mean (SEM; *n* = same as above). *p < 0.05: tPA and prHSP27 vs. tPA, §*p* < 0.001: BSA vs. prHSP27.

There were no significant differences in body weight among the variously treated groups, nor were there significant differences in glucose levels among the groups (Table 1). Ischemic mice were intravenously injected with BSA (*n* = 9), prHSP27 (*n* = 6), tPA (*n* = 7), and tPA plus prHSP27 (*n* = 7) 2 h after the start of reperfusion (Fig 1A). Infarct volumes were measured in cresyl violet-stained sections 24 h after reperfusion (Fig 1B). Brain swelling in each group was 24.9 ± 16.5% in the BSA group, 9.3 ± 7.5% in the prHSP27 group, 31.5 ± 9.8% in the tPA group, and 17.1 ± 10.2% in the tPA plus prHSP27 group, respectively. Brain swelling was decreased by 63% (*p* = 0.016) in the prHSP27 group compared to the BSA group and by 46% (*p* = 0.023) in the prHSP27 plus tPA group compared to the tPA group. tPA did not significantly increase brain swelling compared to BSA (*p* = 0.385) (Fig 1C). Brain infarct volumes (corrected for edema) were 35.6 ± 2.0 mm^3^ in the BSA group, 18.7 ± 4.2 mm^3^ in the prHSP27 group, 38.0 ± 4.6 mm^3^ in the tPA group, and 31.2 ± 4.0 mm^3^ in the tPA plus prHSP27 group (Fig 1D). Infarct volume (corrected for edema) was decreased by 47% (*p* < 0.001) in the prHSP27 group compared to the BSA group and by 18% (*p* = 0.012) in the tPA with prHSP27 group compared with tPA alone. tPA did not significantly increase infarct volume compared to BSA (*p* = 0.247). prHSP27 reduced brain swelling and infarct volume in both groups treated with or without tPA.

**Table 1:** Body weight and blood glucose levels among groups.

Parameter		Substance injected			
BW (g)		BSA	prHSP27	tPA	tPA+prHSP27
	Pre	22.3±1.3	24.6±0.6	23.4±1.2	23.0±1.3
	24 h	19.9±1.0	21.6±0.7	21.3±1.6	20.3±1.6
Blood glucose(mg/dl)					
	Pre	135±11	138±12	147±13	143±11
	15 min	430±118	504±56	496±108	498±34
	1 h	283±53	302±10	285±106	334±20
	2 h	246±44	260±45	272±62	277±66
	3 h	115±22	116±10	111±10	117±12
	4 h	101±7	105±6	100±8	103±13

Body weight and blood glucose levels before surgery (Pre) and at 24 h following reperfusion or at 15 min, 1 h, 2 h, 3 h, and 4 h after D-glucose injection. Data are mean ± SEM (*n* = same as above for all measurements).

Neurological severity scores at 24 h following reperfusion were 8.6 ± 1.1 in the BSA group (*n* = 9), 5.1 ± 1.1 in the prHSP27 group (*n* = 6), 9.1 ± 1.3 in the tPA group (*n* = 7), and 7.6 ± 0.5 in the tPA plus prHSP27 group (*n* = 7) (Fig 1E). prHSP27 decreased neurological severity scores in mice compared to BSA (*p* < 0.001). tPA plus prHSP27 decreased neurological severity scores in mice compared with tPA alone (*p* = 0.014).

### prHSP27 reduces mortality

**Fig 2:**
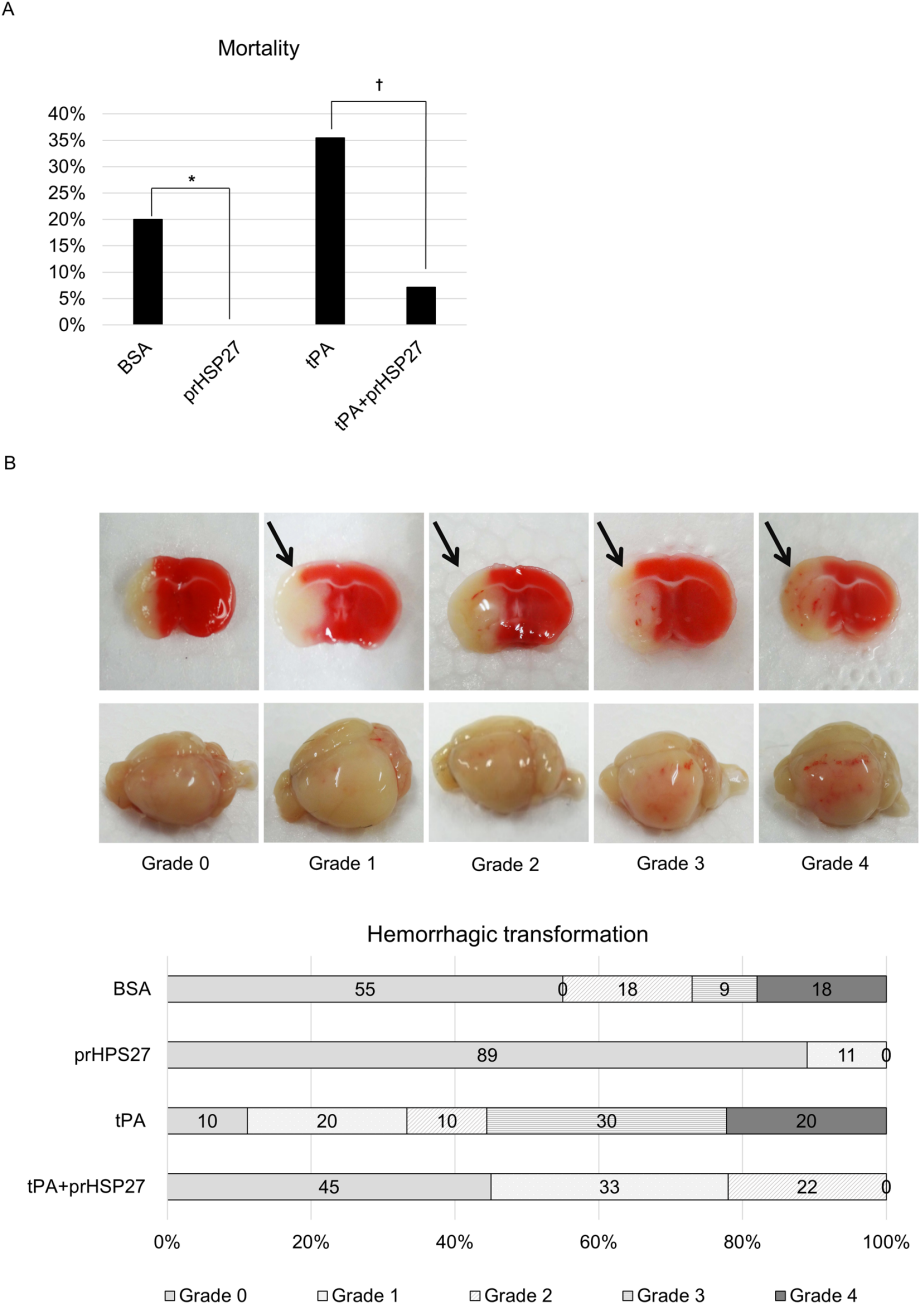
prHSP27 reduces mortality and hemorrhagic transformation at 24 h after reperfusion. (A) At 24 h after reperfusion following MCAO, the mortality rate was 20% (6/30) in the BSA treatment group, 0% (0/15) in the prHSP27 group, 35% (11/31) in the tPA group, and 7% (2/28) in the tPA plus prHSP27 group. *p < 0.05: BSA vs. prHSP27, †*p* < 0.01: tPA plus prHSP27 vs. tPA. (B) Effect of administering tPA on macroscopic hemorrhages. (*Upper*) Representative coronal brain sections stained with 1% 2,3,5-triphenyl tetrazolium chloride at 24 h after reperfusion showing the five grades of hemorrhages: Grades 0–4. (*Lower*) whole, unstained brains with the same hemorrhages.

Mortality rates were 20% in the BSA group, 0% in the prHSP27 group, 35% in the tPA group, and 7% in the tPA plus prHSP27 group (Fig 2A). prHSP27 significantly reduced mortality in mice compared to those injected with BSA. prHSP27 also significantly reduced mortality of mice treated with tPA.

### prHSP27 reduces hemorrhagic transformation

TTC staining of brain sections showed that the prHSP27 plus tPA mouse group (*n* = 9) had macroscopic hemorrhage classifications of grade 0 (*n* = 4; 45%), 1 (*n* = 3; 33%), and 2 (*n* = 2; 22%). By contrast, the tPA group (*n* = 9) had grades of 0 (*n* = 1; 11%), 1 (*n* = 2; 22%), 2 (*n* = 1; 11%), 3 (*n* = 3; 33%), and 4 (*n* = 2; 22%), while the BSA group (*n* = 11) had hemorrhagic grades of 0 (*n* = 6; 55%), 2 (*n* = 2; 18%), 3 (*n* = 1; 9%), and 4 (*n* = 2; 18%) (Fig 2B). Macroscopic hemorrhaging was decreased by 50% (*p* = 0.013) by prHSP27 plus tPA compared to tPA alone and by 27% (*p* = 0.035) by prHSP27 compared to BSA. prHSP27 also reduced the number of mice with major macroscopic hemorrhages of grades 3 and 4. None of the prHSP27-treated mice had grade 3 or 4 macroscopic hemorrhages.

### prHSP27 attenuates blood-brain barrier permeability

**Fig 3:**
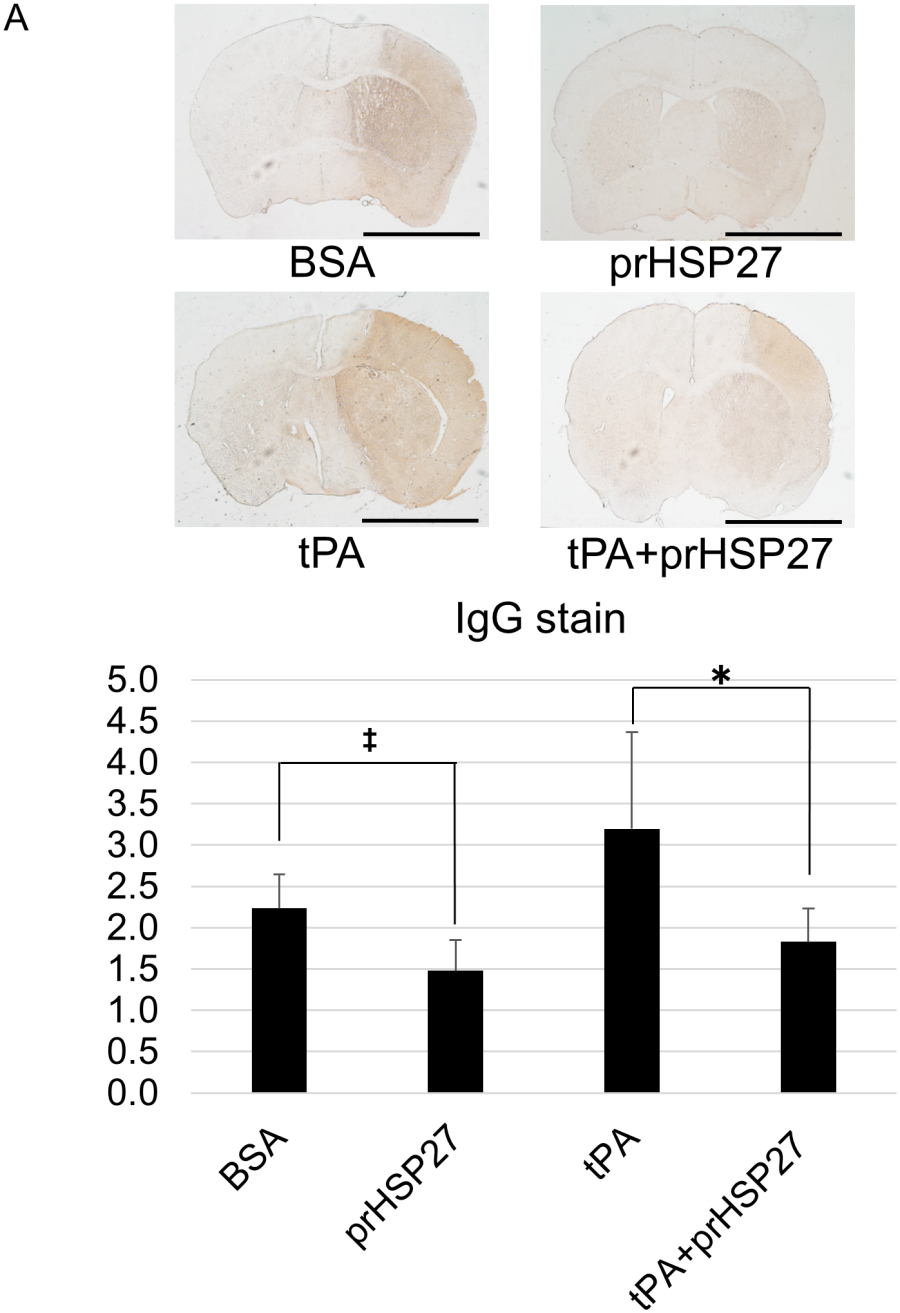
prHSP27 attenuates blood-brain barrier permeability. (A) Representative photomicrographs of endogenous IgG extravasation stained in BSA- (*n* = 7), prHSP27- (*n* = 6), tPA- (*n* = 7), and tPA plus prHSP27- (*n* = 7) treated mice at 24 h after reperfusion. Scale bar = 2 mm. (B) Quantitation of IgG staining in nine consecutive coronal brain slices from each mouse. Data are means ± SEM. *p < 0.05: tPA plus prHSP27 vs. tPA, ‡*p* < 0.005: BSA vs. prHSP27.

We examined the permeability of the BBB by measuring endogenous brain IgG. The density of IgG was 2.28 ± 0.41 in the BSA group (*n* = 7), 1.48 ± 0.37 in the prHSP27 group (*n* = 6), 3.19 ± 1.26 in the tPA group (*n* = 7), and 1.85 ± 0.37 in the tPA plus prHSP27 (*n* = 7) group (Fig 3). IgG extravasation was significantly decreased in the prHSP27 group compared to that in the BSA group (*p* = 0.0038) and significantly decreased in the prHSP27 plus tPA group compared to that in the tPA alone group (*p* = 0.032).

### prHSP27 protects endothelial cells

**Fig 4:**
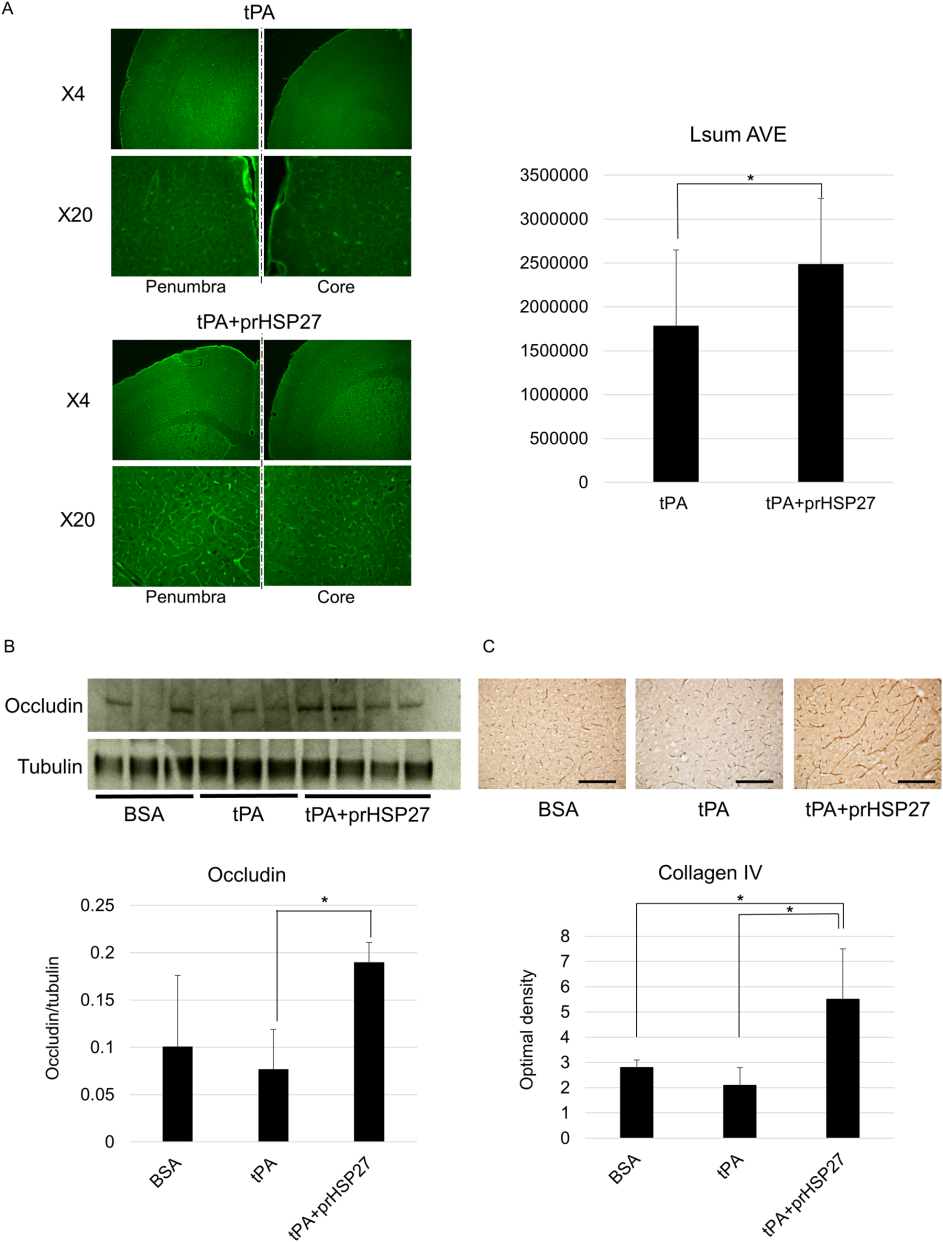
prHSP27 maintains the cell wall structure of endothelial cells and tight junction proteins. (A) Localization of endothelial cell-stained FITC-tomato lectin on the penumbra and core of the ischemic side of the brain from mice treated with tPA or tPA plus prHSP27. FITC-staining is higher in the mice treated with prHSP27. *p < 0.05: tPA vs tPA plus prHSP27. (B) Immunoblot analysis (*upper*) and quantitation (*lower*) of occludin. Protein loading was calculated relative to tubulin. ‡*p* < 0.005: tPA plus prHSP27 vs. tPA. (C) Photomicrographs (*upper*) and quantitation (*lower*) of type IV collagen immunostaining in the infarct boundary zones in BSA- (*n* = 5), tPA- (*n* = 5), and tPA plus prHSP27- (*n* = 5) treated mice at 24 h after reperfusion. prHSP27 maintained the type IV collagen structure of the basement membrane. Scale bars = 100 μm. Data are mean ± SEM. *p < 0.05: tPA plus prHSP27 vs. BSA, tPA plus prHSP27 vs. tPA.

As the disruption of the BBB due to the combined induction of hyperglycemia and tPA was equal to or greater than that induced by hyperglycemia alone, we continued to use the combined induction for all further studies. The observation that prHSP27 reduced brain edema and macroscopic hemorrhages suggested that it might have preserved the structure of endothelial cells of brain arteries in the ischemic boundary zone of these mice. We evaluated endothelial cell wall images stained with FITC-tomato lectin. FITC staining was significantly higher in the mice treated with tPA plus prHSP27 than those treated with tPA alone (*p* = 0.021) (Fig 4A), indicating that prHSP27 maintained the cell wall structure of endothelial cells. prHSP27 also increased the levels of occludin in hyperglycemic tMCAO mice treated with tPA vs. those treated with tPA alone (*p* = 0.028) (Fig 4B). The optical density of collagen IV, a basement membrane protein, was not significantly changed by tPA treatment compared to BSA treatment (*p* = 0.091), while in the mice treated with tPA, prHSP27 increased the levels of collagen IV (*p* = 0.016) (Fig 4C).

### prHSP27 reduces MMP-9 activity and microglial activation

**Fig 5:**
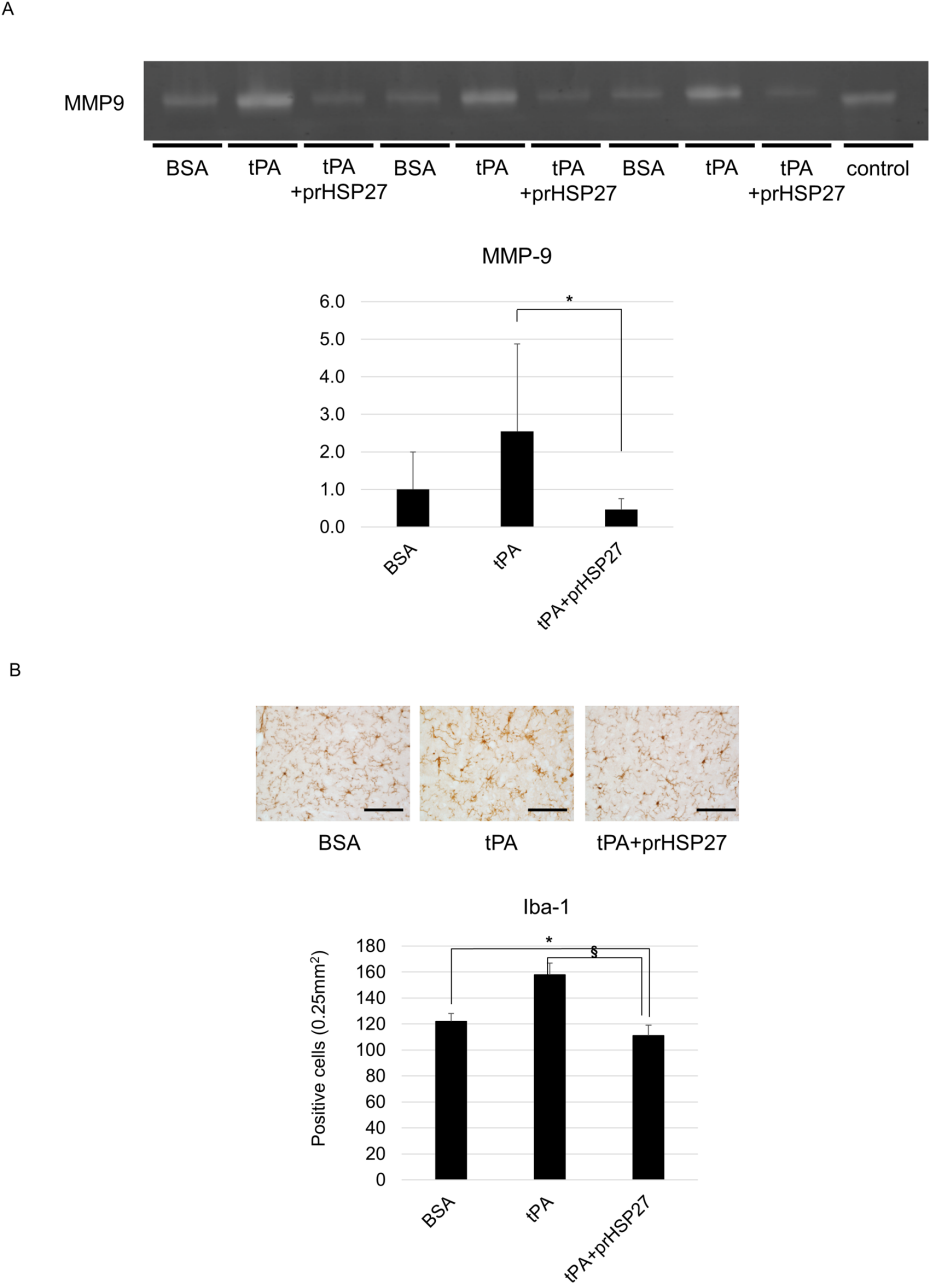
Effect of prHSP27 on the expression of matrix metalloproteinase-9 (MMP-9) on the ischemic side. (A) Zymographic (upper) and densitometric (lower) analyses of MMP-9 protein in BSA- (*n* = 7), tPA- (*n* = 7), and tPA plus prHSP27- (*n* = 7) treated mice at 24 h after reperfusion. ‡*p* < 0.005: tPA plus prHSP27 vs. tPA. (B) Photomicrographs of ionized calcium binding adapter molecule-1 (Iba-1) immunostaining (*upper*) and number of Iba-1-positive cells (*lower*) in the infarct boundary zones in BSA- (*n* = 5), tPA- (*n* = 5), and tPA plus prHSP27- (*n* = 5) treated mice at 24 h after reperfusion. prHSP27 suppressed the inflammatory response. Scale bars = 100 μm. Data are mean ± SEM. *p < 0.05: tPA plus prHSP27 vs. BSA, ^**§**^*p* < 0.001: tPA plus prHSP27 vs. tPA.

We measured the activity of MMP-9, which degrades collagen type IV, by gelatin zymography (Fig 5A). In hyperglycemic tMCAO mice, tPA tended to increase the activity of MMP-9, and prHSP27 attenuated this increase (*p* = 0.03). To evaluate the inflammatory microglial activation, we counted the Iba-1-positive microglia. In the ischemic boundary zone, the number of cells immunopositive for Iba-1 was significantly lower in the hyperglycemic tMCAO mice injected with prHSP27 plus tPA than in those with rtPA alone (*p* < 0.001) (Fig 5B).

### prHSP27 preserves BBB structure

**Fig 6:**
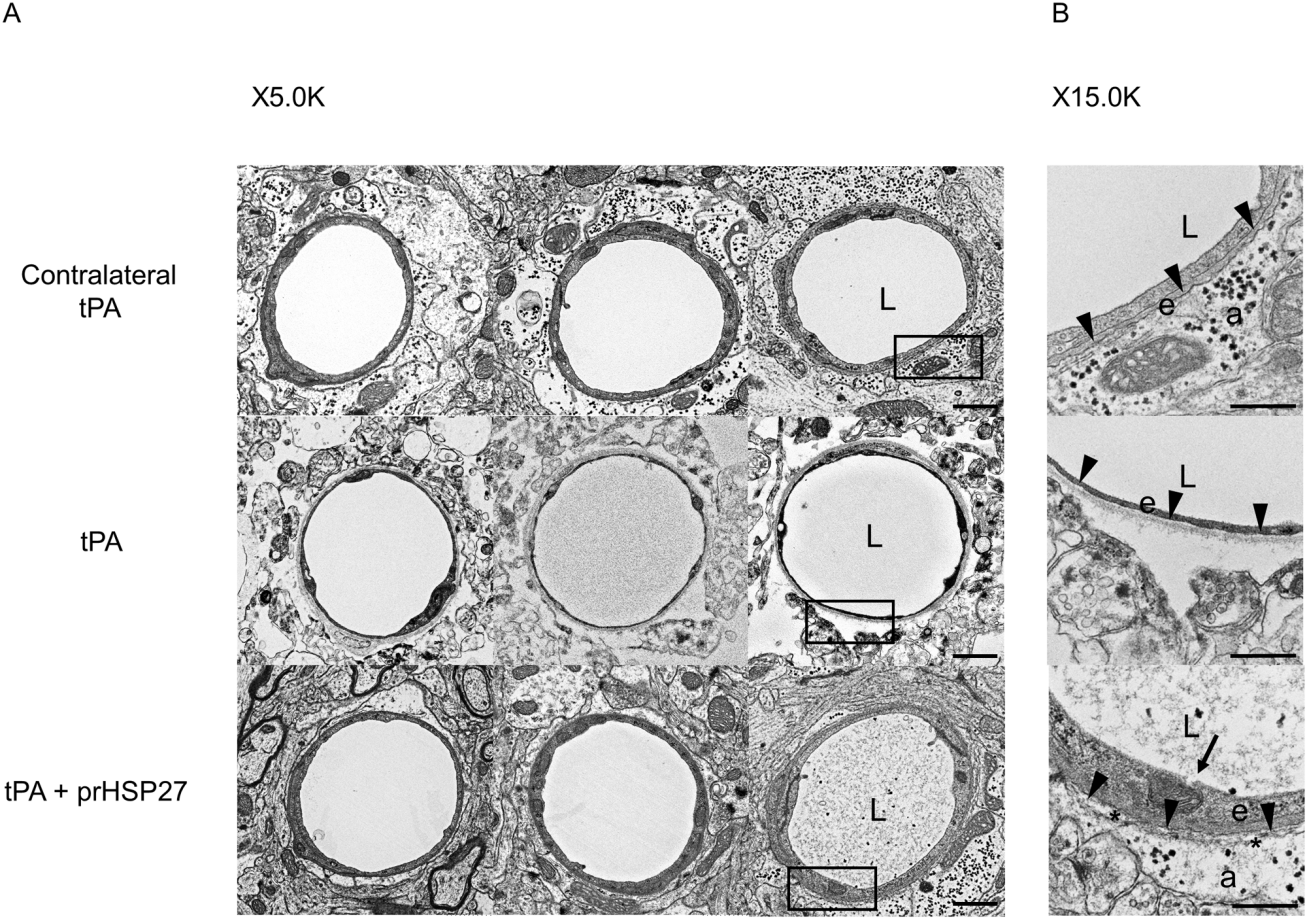
Effect of cerebral ischemia on prHSP27 in the blood-brain barrier. **(A)** Electron microscopic analysis of the blood-brain barrier on the non-ischemic side of tPA- (contralateral tPA, n = 3), ischemic side of tPA- (tPA, n = 3), and ischemic side of tPA plus prHSP27- (tPA and prHSP27, n = 3) treated mice. Magnification, ×5000 (*left row*) and ×15000 (*right row*). The left row shows magnifications of the boxed areas in the right row. Scale bars, 1 μm (*left row*) and 500 nm (*right row*). a: astrocyte endfeet; e: endothelial cell; L: lumen. Arrowheads: the space between the basement membrane and astrocyte endfeet. Asterisks indicate the space between the basement membrane and astrocyte endfeet (B).

Finally, we observed changes in BBB structure via electron microscopy. In the tPA-treated mice, the basement membrane was thinner and there was more detachment between astrocytes and the basement membrane on the ischemic side of the brain than on the non-ischemic side. When prHSP27 was injected along with tPA, basement membranes were thicker and there was less detachment between astrocytes and basement membranes (Fig 6), suggesting that prHSP27 preserved BBB structure against ischemic insult in the presence of hyperglycemia and tPA.

## Discussion

In the present study, we examined and confirmed the brain protective effects of intravenously injected prHSP27 in hyperglycemic tMCAO mice treated with tPA, which enhance the BBB-disruption. We first verified that prHSP27 decreased brain edema and infarct volume and improved neurological symptoms in hyperglycemic tMCAO mice in the presence or absence of tPA, thus confirming the effectiveness of prHSP27 against ischemic injury [18,19], and showing that prHSP27 was also effective against ischemic brain damage in mice whose BBB was severely injured. Therefore, we have now demonstrated the effectiveness of the combination therapy of prHSP27 and tPA.

Second, we showed that prHSP27 attenuated the BBB damage in this model. prHSP27 decreased both hemorrhagic transformation, especially parenchymal macroscopic hemorrhages, and BBB permeability in hyperglycemic tMCAO mice treated with tPA. Furthermore, prHSP27 decreased MMP-9 activity, which tended to be increased by tPA, and preserved endothelial cell walls. Electron microscopic studies also showed that prHSP27 preserved BBB structure on the ischemic side of brains in tPA-treated mice. Together, these findings argue strongly in favor of a protective role of prHSP27 in maintaining the BBB and suggest the possibility of using prHSP27 therapy in patients with stroke.

Leak et al. also showed that BBB disruption caused by ischemic injury due to tMCAO was attenuated in mice over-expressing transgenic HSP27 [37]. There are two major differences between their study and ours. One, while they used only ischemic insult, we used a triple method (hypoglycemia, tMCAO, and tPA) to injure the BBB, and two, while transgenic HSP27 was overexpressed before the ischemic insult in Leak’s studies, we injected HSP27 after the ischemic and hyperglycemic insults, which is closer to the situation that would be experienced by stroke patients.

We consider that prHSP27 preserves endothelial cells, glia, and neurons both intracellularly and extracellularly. Previously, we showed that some injected prHSP27 is found in neurons [18]. We speculate that small amounts of prHSP27 might also localize in endothelial cells and glia. We hypothesize that intracellular prHSP27 preserved endothelial cells, glia, and neurons by inhibiting the apoptotic pathway, preventing aggregations of misfolded proteins, and stabilizing the cytoskeleton, and attenuated ischemic brain damage and BBB damage in this study. prHSP27 was injected intravenously, suggesting that it might have first affected endothelial cells and preserved them, and then subsequently protected other cells. These hypotheses remain to be tested. Recently, the functions of extracellular HSP27 were reported [38–40]. Extracellular HSP27 was also thought to function as an immunomodulatory molecule [39,41]. Brain ischemia induces an immunological response, microglial activation, and subsequent recruitment of circulating leukocytes to the ischemic brain [42,43]. We demonstrated that prHSP27 decreased the number of cells immunopositive for Iba-1, a marker of inflammation, suggesting that it eventually attenuates and modulates immune responses. prHSP27 may protect endothelial cells, glial cells, and neurons both intracellularly and extracellularly, thereby maintaining BBB function.

We demonstrated the possibility of co-administering tPA and prHSP27 for stroke treatment. tPA is the only beneficial pharmacotherapy for acute ischemic stroke, but also injures the BBB and increases the risk of brain edema and secondary hemorrhagic transformation. prHSP27 decreased not only infarct volume but also hemorrhagic transformation, a major complication of tPA therapy. These results suggest that combination therapy using tPA with prHSP27 may achieve better outcomes in acute cerebral ischemia. prHSP27 might be a useful therapeutic agent to protect against not only acute cerebral ischemic stroke but also acute hemorrhagic stroke.

We found that prHSP27 had no effect on blood glucose, the inducer of hemorrhagic transformation in our model, indicating that the decreased hemorrhagic transformation and BBB disruption were independent of their effects on glycemic control. The 2-mg/kg-dose of prHSP27 required for neuroprotection in our model appears to be adequate for its protective effects. We injected prHSP27 intravenously, that should be the same administration route used clinically. Further studies are needed to establish them (Guidance for Industry Estimating the Maximum Safe Starting Dose in Initial Clinical Trials for Therapeutics in Adult Healthy Volunteers, http://www.fda.gov/downloads/Drugs/Guidances/UCM078932.pdf#search=%27guidekines+for+industry+sfe+starting%27).

We think that prHSP27 might be a suitable combination therapy for patients with ischemic stroke who have been administrated or require tPA therapy. prHSP27 might decrease infarct volume and prevent secondary hemorrhage caused by tPA injection. Recently, rHSP27 administration was reported to be effective in other disease model animals. Subcutaneous injection of rHSP27 reduced atherosclerotic lesions in mice and humans [44], and implantation of rHSP27-eluting stents in rabbit carotid arteries markedly improved re-endothelialization in an artery injury model [17]. Thus, the possibility of HSP27 administration therapy is growing and might be useful in future stroke and other disease therapy.
